# Impact of resistance training on the autophagy-inflammation-apoptosis crosstalk in elderly subjects

**DOI:** 10.18632/aging.101167

**Published:** 2017-02-02

**Authors:** Yubisay Mejías-Peña, Brisamar Estébanez, Paula Rodriguez-Miguelez, Rodrigo Fernandez-Gonzalo, Mar Almar, José A. de Paz, Javier González-Gallego, María J. Cuevas

**Affiliations:** ^1^ Institute of Biomedicine (IBIOMED), University of León, León, Spain; ^2^ Division of Clinical Translational Science, Georgia Prevention Institute, Department of Pediatrics, Augusta University, USA; ^3^ Radiobiology Unit, Laboratory of Molecular and Cellular Biology, Institute for Environment, Health and Safety, Belgian Nuclear Research Centre (SCK·CEN), Mol, Belgium

**Keywords:** elderly, autophagy, inflammasome, apoptosis, resistance exercise

## Abstract

Aging is associated with a decline in autophagy and a state of low-grade inflammation which further affects apoptosis and autophagy. Importantly, these alterations could reverse with regular physical activity. This study assessed the effects of a resistance exercise training program on autophagy, NLRP3 inflammasome, and apoptosis in peripheral blood mononuclear cells (PBMCs) from old subjects. Twenty-six healthy women and men (age, 69.6±1.5 yr) were randomized to a training (TG) or a control (CG) group. TG performed an 8-week resistance training program, while CG followed their daily routines. Protein expression of beclin-1, Atg12, Atg16 and LAMP-2 increased following the training program, while expression of p62/SQSTM1 and phosphorylation of ULK-1 at Ser757 were significantly lower. Resistance exercise also induced a decrease in NLRP3 expression and in the caspase-1/procaspase-1 ratio. Expression of Bcl-2 and Bcl-xL, as well as the Bad/BcL-2 ratio were reduced, and there was a significant decrease in the protein content of caspase-3. The results obtained seem to indicate that 8-week resistance training stimulates autophagy, prevents NLRP3 inflammasome activation, and reduces apoptosis in PBMCs from elderly subjects. These data could have a significant impact in prevention and rehabilitation programs currently employed in elderly population.

## INTRODUCTION

Autophagy is a catabolic process of eukaryotic cells, contributing to the degradation in lysosomes of unwanted or damaged components [1]. Autophagy proceeds through a series of cellular events, including induction of the formation of autophagic isolated membranes (called phagophore), elongation of the phagophore and sequestration of cytosolic components through formation of the autophagosome, transport to the lysosome, degradation, and use of the products of degradation [2]. The nucleation of the autophagosomal membrane is controlled by a molecular complex containing Bcl-2-interacting protein (beclin)-1 [3]. Autophagosome formation is under the control of autophagy related genes (Atg). Specifically, two systems of conjugation similar to ubiquitin have been described. The first, the Atg12-Atg5 complex interacts with Atg16 to participate in the formation of autophagosome membrane. Atg16 has also been postulated as the gene linking autophagy and inflammation in several pathologies [4]. The second system involves the mammalian protein 1 light chain 3 (LC3), with formation of the LC3II isoform, which then binds to the adaptor protein p62 sequestrosome 1 (p62/SQSTM1), facilitating the autophagic degradation of ubiquitinated protein aggregates in lysosomes [5].

Autophagy plays an important role in several physiological processes, and autophagic dysfunctions cause great challenges in the cell. This can result in different diseases, many of which worsen with age [6]. Since a common feature of cell aging is the accumulation of damaged proteins and organelles, autophagy has been proposed as a key function in regulating senescence and longevity, but unfortunately this process becomes less efficient with age [7]. However, regular physical exercise has been identified as an inducer of autophagy *in vivo*, delaying the onset of aging and associated comorbidities [8]. Supporting this idea, previous results from our research group have shown that an aerobic exercise training program induced a higher expression of several markers of autophagy in peripheral blood mononuclear cells (PBMCs) from elderly subjects [9].

An age-related decline in autophagic degradation seems to occur concomitantly with a low-grade systemic inflammation. In fact, impaired autophagy can trigger the appearance of a pro-inflammatory phenotype tissue (or *inflammaging*) and the inflammasome activation [10, 11]. Sedentary lifestyle, like age, is associated with an elevation of inflammatory markers, and chronic exercise can exert a powerful anti-inflammatory effect [12]. Indeed, cross-sectional studies have confirmed an association between physical inactivity and moderate degree of inflammation in healthy individuals [13] and in the elderly population [14]. In addition, regular physical activity induces an attenuation of biomarkers of inflammation and toll-like receptor (TLR) signalling pathways in old subjects, suggesting that physical exercise could suppress moderate systemic inflammation in the elderly [15, 16]. However, other specialized innate immune receptors, such as the NOD-like receptor family, pyrin domain containing 3 (NLRP3), seem to mediate chronic low-level inflammation during aging and in aging-related pathologies [17]. In particular, NLRP3, a key component of the inflammasome, is an important regulator of age-related inflammation [18]. Interestingly, emerging evidence suggest that NLRP3 inflammasome activation may be inhibited by physical exercise [19].

Apart from a decline in autophagy and a low-grade inflammatory phenotype, another hallmark of the aging process is an increased apoptosis, which negatively correlates with autophagy. It seems likely that increased expression of anti-apoptotic Bcl-2/xL proteins represses beclin-1-dependent autophagy, which triggers the expression of cell cycle inhibitors and induces cellular senescence [11]. In addition, the interaction between the anti-apoptotic Bcl-2 protein and beclin-1 appears to be an important regulator of autophagy induced by exercise [20, 21].

To this background, this study investigated the potential of an 8-week resistance training program to prevent age-related decline in autophagy, chronic low-grade inflammation and apoptosis in PBMCs from old subjects. Since aging is associated with a decline in autophagy and the appearance of a low-grade inflammation, which further affects apoptosis and autophagy, and considering that these alterations may reverse with regular physical activity, we hypothesize that resistance exercise training would activate autophagy and reduce the inflammation and apoptosis associated to age.

## RESULTS

There were no differences between training group (TG) and control group (CG) in age, height, body weight, and body mass index before and after the intervention. All participants from TG showed a 100% compliance with the training protocol. Maximal voluntary isometric contraction (MVIC) in leg press (100.0 ± 10.2 *vs*. 135.7 ± 11.9 kg; p <0.05) and in biceps curl bench (12.8 ± 0.9 *vs*. 18.5 ± 1.7 kg; p <0.05) increased in TG from pre to post. Similarly, the resistance training increased one repetition maximum (1RM) in leg press (150.0 ± 12.5 *vs*. 195.8 ± 12.7 kg; p <0.05) in biceps curl bench (20.1 ± 1.3 *vs*. 26.4 ± 2.3 kg; p <0.05) and in a seated pec deck (27.5 ± 0.8 *vs*. 33.5 ± 1.7 kg; p <0.05). CG showed no functional changes between before and after the intervention period.

### Effect of resistance exercise training on autophagy signaling pathways

**Figure 1:**
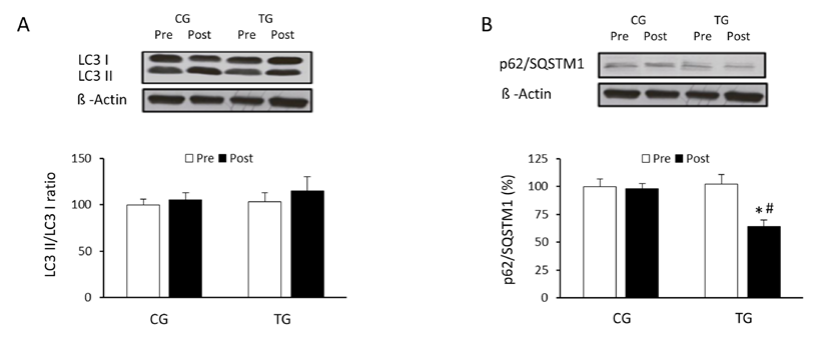
Effects of resistance training on LC3I, LC3II and p62/SQSTM1 expression Representative Western blots and densitometric quantification of LC3II, LC3I (**A**) and p62/SQSTM1 (**B**) in response to 8 weeks of resistance training for TG and the same period of normal daily routines for CG. Protein from PBMCs was separated by sodium dodecyl sulfate-polyacrylamide gel electrophoresis, followed by immunoblotting. Equal loading of proteins is illustrated by β-actin bands. Values are means ± SEM.*p<0.05 *vs.* CG; #p <0.05 *vs.* Pre within a group.

**Figure 2:**
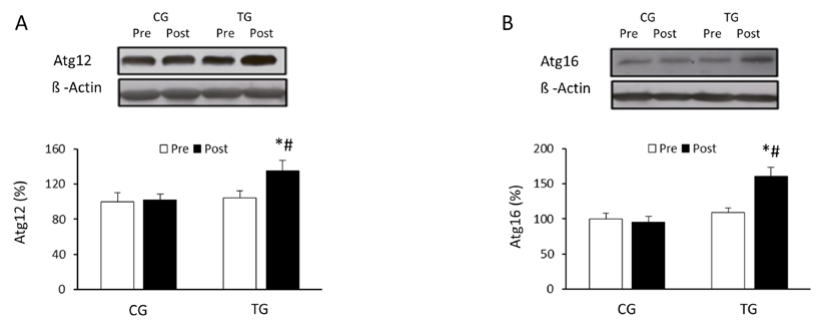
Effects of resistance training on Atg12 and Atg16 expression Representative Western blots and densitometric quantification of Atg12 (**A**) and Atg16 (**B**) in response to 8 weeks of resistance training for TG and the same period of normal daily routines for CG. Protein from PBMCs was separated by sodium dodecyl sulfate-polyacrylamide gel electrophoresis, followed by immunoblotting. Equal loading of proteins is illustrated by β-actin bands. Values are means ± SEM.*p<0.05 *vs*. CG; #p <0.05 *vs.* Pre within a group.

**Figure 3:**
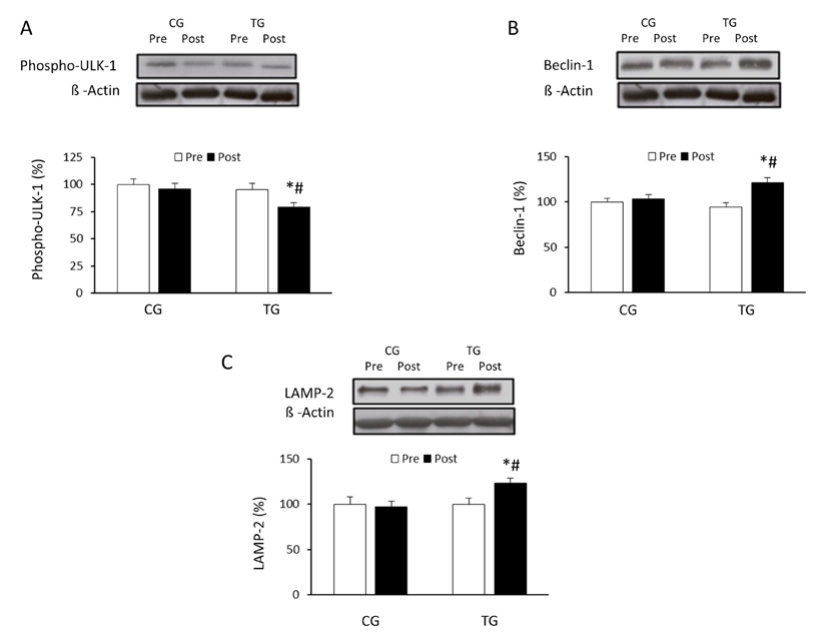
Effects of resistance training on Ser757 phospho-ULK-1, beclin-1 and LAMP-2 expression Representative Western blots and densitometric quantification of Ser757 phospho-ULK-1 (**A**), beclin-1 (**B**) and LAMP-2 (**C**) in PBMCs in response to 8 weeks of resistance training for TG and the same period of normal daily routines for CG. Protein from PBMCs was separated by sodium dodecyl sulfate-polyacrylamide gel electrophoresis, followed by immunoblotting. Equal loading of proteins is illustrated by β-actin bands. Values are means ± SEM.*p<0.05 *vs*. CG; #p<0.05 *vs*. Pre within a group.

The effects of exercise on the autophagic flux were investigated in PBMCs of the elderly after 8 weeks of resistance training or daily routines. Resistance training induced a non-significant increase in the LC3II/LC3I ratio (p =0.063) whereas CG values did not change (p =0.125) (Figure 1A). p62/SQSTM1 protein level was significantly reduced (p <0.05) in response to resistance exercise, reaching values significantly lower than those from CG after the training period (Figure 1B). Regarding Atg12 (Figure 2A) and Atg16 (Figure 2B), a significant increase in protein content was observed in TG when compared with both pretraining (Atg12, p <0.03 and Atg16, p <0.02, respectively) and CG values after 8 weeks of exercise training (Atg12, p <0.03 and Atg16, p <0.02, respectively). The current training program had opposite effects on the protein content of ULK-1 phosphorylated at serine 757 and beclin-1 (Figure 3A and 3B). While phospho-ULK-1 significantly decreased (p <0.05) after completion of training, beclin-1 increased significantly (p <0.05) as a result of the 8 weeks of training. Phosphorylation of ULK-1 was also significantly lower (p <0.05) and beclin-1 protein concentration significantly higher (p <0.05) in TG than in CG after the intervention period. The resistance training program significantly increased the content of lysosomal protein LAMP-2 compared with pretraining values (p<0.05) and GC after the 8-week intervention (p<0.05) (Figure 3C).

### Effect of resistance exercise on NLRP3 inflammasome components and activation

**Figure 4:**
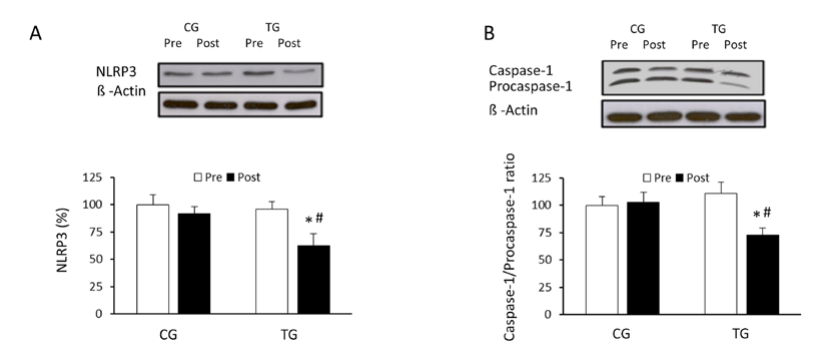
Effects of resistance training on NLRP3 inflammasome expression and caspase-1/procaspase-1 ratio Representative Western blots and densitometric quantification of NLRP3 (**A**), caspase-1 and procaspase-1 (**B**) in PBMCs in response to 8 weeks of resistance training for TG and the same period of normal daily routines for CG. Protein from PBMCs was separated by sodium dodecyl sulfate-polyacrylamide gel electrophoresis, followed by immunoblotting. Equal loading of proteins is illustrated by β-actin bands. Values are means ± SEM.*p<0.05 *vs*. CG; #p<0.05 *vs*. Pre within a group.

To analyze the effects of resistance training on the expression of the components of the inflammasome and its state of activation in PBMCs of old subjects, protein concentration of NLRP3, pro-caspase-1 and caspase-1 were analysed, as well as the caspase-1/procaspase-1 ratio. After the 8-week training, NLRP3 protein levels decreased significantly in the trained group compared to pretraining values and to CG values at post (p <0.03 and p <0.04, respectively) (Figure 4A). A training effect was observed in the caspase-1/procaspase-1 ratio (Figure 4B); the relative ratio decreased significantly (p <0.04) in response to the resistance exercise protocol.

### Effect of resistance exercise on apoptosis-related proteins

**Figure 5:**
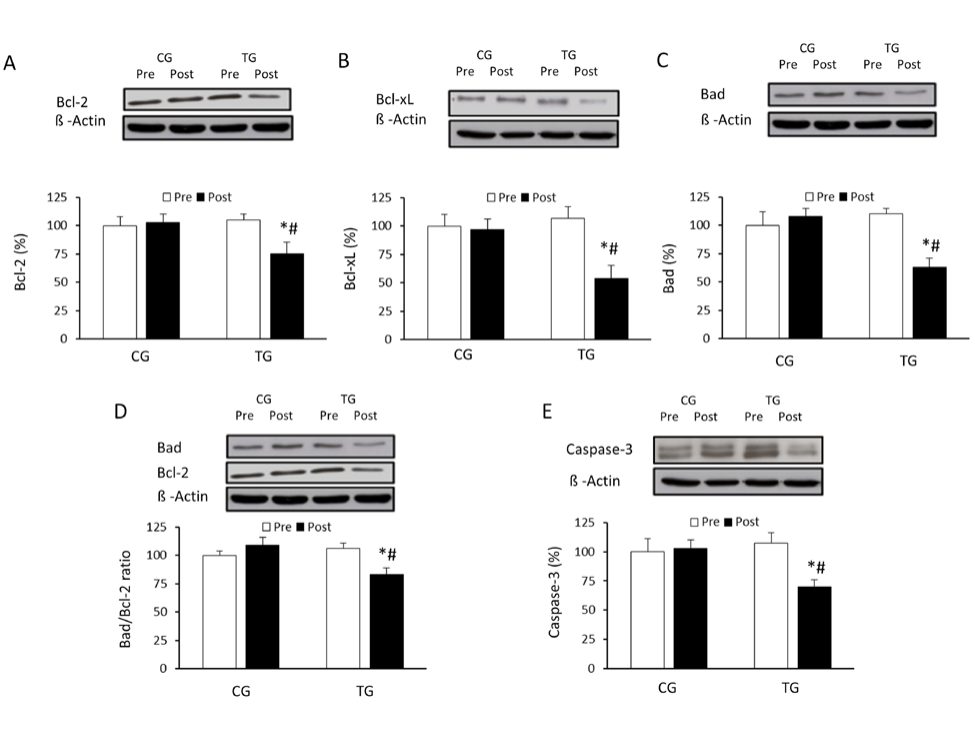
Effects of resistance training on BcL-2, Bcl-xL, and Bad expression and cleaved caspase-3 Representative Western blots and densitometric quantification of Bcl-2 (**A**), Bcl-xL (**B**), and Bad (**C**), Bad/Bcl-2 ratio (**D**) and cleaved caspase-3 (**E**) in PBMCs in response to 8 weeks of resistance training for TG and the same period of normal daily routines for CG. Protein from PBMCs was separated by sodium dodecyl sulfate-polyacrylamide gel electrophoresis, followed by immunoblotting. Equal loading of proteins is illustrated by β-actin bands. Values are means ± SEM.*p<0.05 *vs*. CG; #p<0.05 *vs*. Pre within a group.

To analyze the effect of resistance training in PBMCs from elderly subjects apoptosis processes, and its relationship with autophagy, the concentrations of the anti-apoptotic Bcl-2 and Bcl-xL proteins were measured. As illustrated in Figure 5A and 5B, concentration of these anti-apoptotic proteins decreased significantly in the trained group compared to the pretraining values (p <0.05 for Bcl-2 and p <0.04 for Bcl-xL, respectively), resulting in significant differences between TG and CG after the intervention (p <0.05 for Bcl-2 and p <0.04 for Bcl-xL, respectively). The training protocol also induced a significant downregulation in one of the most representative pro-apoptotic proteins, Bad (p <0.04) (Figure 5C). The significant decrease observed in the relative Bad/Bcl-2 ratio (p <0.05) (Figure 5D) suggested a decrease of the apoptotic process after 8 weeks of resistance exercise. In this line, caspase-3 was also substantially decreased when compared to basal conditions (p <0.05) and control group (p <0.05) (Figure 5E).

## DISCUSSION

The current study shows that an 8-week resistance training program induces an activation of the autophagy machinery in PBMCs from elderly subjects. In addition exercise-induced autophagy adaptations are accompanied by a downregulation of the NLRP3 inflammasome and the apoptotic processes.

Accumulating evidence reports a trend towards a decreased autophagy in different organs and tissues, including peripheral mononuclear cells from elderly subjects, when compared to young volunteers [9, 22]. Among the interventions to prevent or reverse the unwanted effects of age, including the autophagic decline, exercise seems to be among the most effective [8]. In fact, chronic aerobic exercise promotes autophagy in human skeletal muscles and lymphocytes 5, 9, 23, 24]. Interestingly, this type of training induces autophagy especially in glycolytic muscles when the adaptive changes aimed at the generation of oxidative phenotype are still ongoing [25]. On the other hand, chronic resistance training also stimulates the autophagic machinery in the gastrocnemius muscles of aged rats [26]. Up till now however, there was no information showing such adaptations in human PBMCs.

After completing the resistance training program, we found only a slight and non-significant increase in the ratio LC3II/I. Previous data indicate that the conversion of LC3I to LC3II was depressed in skeletal muscle following an acute bout of resistance exercise in young and older participants [27], suggesting a decrease in autophagy. Likewise, 9 weeks of resistance training decreased the LC3II/I ratio in skeletal muscle of aged rats [26]. However, another study did not show any variation of LC3II protein content 1 h after resistance exercise in vastus lateralis muscle from young healthy males subjects [28]. It is important to note that the LC3II/LC3I ratio is involved in early and late stages of autophagic flux, from the formation of the autophagosome to the degradation in the lysosome. Therefore, other markers should be evaluated in order to assess exercise-induced changes in the autophagy process [24, 29].

Results from the current study indicate that resistance exercise induced a significant decrease in the expression of p62/SQSTM1. These data support past research in aged rats [26], and could suggest a possible increase in autophagy after the training program. However, the content of this protein is not always inversely correlated with autophagic activity [30]; although p62 is degraded in the process of autophagy and accumulates when this process is inhibited, it is possible that increased production of p62 equals the increment in p62 degradation during exercise. Therefore, to further understand the activation of the autophagic machinery induced by physical exercise, the expression of different proteins promoting the initial assembly of the autophagosome membrane were also measured in the current investigation. The first ubiquitin conjugation system involved in the formation of the autophagosome is Atg12/Atg5/Atg16 [31]. Results from our study indicate that the expression of Atg12 and Atg16 protein increased significantly in old subjects after the resistance training program. In addition, exercise also affected other proteins of the autophagic pathway. Specifically, a significant reduction in phosphorylation of ULK-1 at serine 757 and increased expression of beclin-1 was detected. ULK-1 plays a critical role in the initiation of autophagy. In fact, ULK-1 phosphorylation on serine residue 555 is an important stimulus to activate autophagy, whereas phosphorylation at serine 757 has an inhibitory effect [32]. The significant decrease in the ULK-1 phosphorylated at serine 757 reinforces the fact that resistance training stimulates autophagy. These results are similar to those reported after aerobic training in old subjects [8]. Another key protein in the autophagic process is beclin-1 [25]. In the present study, the significant increase detected in the content of beclin-1 in the trained group is consistent with previous studies that examined the effects of chronic resistance training in older rats [26], and further supports the hypothesis that regular exercise could have a preventive effect on the loss of efficiency of the autophagic process associated with aging. Moreover, these results, together with previous data from our research group, suggest that changes in autophagy could be a common response to any form of regular exercise [9].

In a final step, the lysosome associated membrane protein 2a (LAMP-2) plays a critical role [9]. The attenuation of the autophagy machinery due to aging includes a reduction on LAMP-2 levels in both oxidative and glycolytic muscles, suggesting a declined ability to eliminate damaged or inefficient organelles [25]. In the current study, the concentrations of LAMP-2 increased after resistance training when compared to basal values in elderly subjects. This result concords with previous research showing a tendency to increase LAMP-2 mRNA levels in response to a 6-month weight loss program combined with life-long voluntary exercise in older overweight women [33].

Recent research has established that autophagy is heavily linked to immune cell responses and inflammatory processes [34], mitigating or suppressing the inflammatory response [35]. On one hand, autophagy negatively regulates inflammation by inducing changes on the TLR-mediated inflammatory response, as we recently showed [9]. On the other hand, autophagy may function as a negative counter-regulatory mechanism for inflammasome activation, thus providing a checkpoint to limit the development of inflammation [36]. In fact, aging is accompanied by a state designated *inflammaging*, which usually correlates with NLRP3 inflammasome activation or autophagy decline [37]. By preserving the balance between these two processes (i.e., decreasing NLRP3 inflammasome activation and increasing autophagy), inflammation can be maintained at physiologically normal levels. The present study shows that NLRP3 inflammasome levels are reduced after resistance training in old subjects. These results are supported by previous reports of inflammasome expression reduction and systemic downregulation of inflammatory cytokines in adipose tissues from rats subjected to regular endurance or resistance exercise training [38].

NLRP3 inflammasome is composed of NLRP3, the adaptor molecule apoptotic speck-containing protein with a card (ASC), and procaspase-1. Activated caspase-1 in turn cleaves >70 substrates, including the proinflammatory cytokines interleukin-1 (IL-1) and IL-18. Emerging evidence suggests that pro-inflammatory cytokines IL-1 and IL-18 show an age-dependent regulation, which seems to implicate inflammasome-mediated caspase-1 activation in the aging process [18]. In the present investigation, the resistance training program alleviated the activity of caspase-1 and the cleavage of procaspase-1 (caspase-1/procaspase-1 ratio decreased) through inhibition of the NLRP3 inflammasome activation. The resistance training program employed here has been reported to reduce the expression of inflammatory markers [15] and with the current data, we suggest that it also induces autophagy, and decreases NLRP3 activation. Overall, the proinflammatory phenotype associated with aging seems to be related to the autophagic state, and both are attenuated in response to resistance exercise training.

Autophagy is important in the repairment of cellular materials, as well as in self-killing of irreversibly injured cells. In fact, programmed cell death (PCD) is also classified into PCD1 (apoptosis) and PCD2 (autophagic cell death) [39]. Thus, autophagy and apoptosis are crucial cellular housekeeping and tissue survival mechanisms. Moreover, an age-related decline in autophagic degradation has been shown to occur concomitantly with age-related increases in apoptosis, which negatively correlates with autophagy [8]. The beclin-1 protein appears to be able to control all these aspects but, currently, there are only indirect evidences about its role in the regulation of aging [40]. On the other hand, Bcl-2 family members act as regulators of the cell cycle, but also control autophagy. Anti-apoptotic proteins such as Bcl-2 and Bcl-xL can inhibit autophagy, while proapoptotic proteins, such as Bad, can induce it. It has been suggested that Bcl-2 inhibits autophagy through a direct interaction with the BH3 domain of the autophagy protein beclin-1 at the endoplasmic reticulum [41]. Therefore, it seems likely that the increased expression of anti-apoptotic Bcl-2/xL proteins represses beclin-1-dependent autophagy, which triggers the expression of cell cycle inhibitors and induces cellular senescence [11]. In addition, it seems that the age-related increase in apoptosis could be attenuated by caloric restriction and exercise, perhaps through autophagy induction [8]. Findings from the current study show that resistance exercise induced an increase in beclin-1, which was accompanied by decreased Bcl-2 and Bcl-xL protein concentration. It is important to note that several authors have shown that the expression of Bcl-2 and Bcl-xL increases with aging in many tissues [11,42]. Furthermore, the relative Bad/Bcl-2 ratio, a parameter of apoptotic cell death, decreased with the training program. Our results confirm previous data showing that exercise-induced autophagy *in vivo* involves a disruption of the Bcl-2–beclin-1 complex [20]. In fact, the disruption of the Bcl-2-beclin-1 complex is crucial to induce autophagy in mammalian cells [20,21]. Moreover, other studies in humans also reported that the benefits of resistance exercise training are associated with increased autophagy activity and reduced apoptosis in aged skeletal muscles [26]. The present results suggest that exercise-induced autophagy in PBMCs from old subjects could inhibit the direct interaction between Bcl-2/xL and beclin-1, avoiding the breakdown of the beclin-1/VSP34 complex and activating its interaction with other positive regulators of autophagy [41,43]. Moreover, the trained group of the current investigation showed reduced apoptosis, as assessed by caspase-3, after the intervention. These results are in accordance to the decreased caspase-3 mRNA content observed in the gastrocnemius of trained old rats [44]. Human studies also indicate that an intense exercise lifestyle was able to attenuate the effects of aging on caspase-3 expression in T cells [45]. Thus, it appears that autophagy induction and reduced apoptosis may contribute to the beneficial metabolic effects of exercise during aging. Moreover, inflammasomes could also contribute to this interplay between apoptosis and autophagy, given that the inflammasome receptors can directly interact with beclin-1 [46].

In summary, our results seem to indicate that 8 weeks of resistance exercise training stimulates autophagy, prevents NLRP3 inflammasome activation and reduces apoptosis in PBMCs from elderly subjects. These novel data suggest that the well-established crosstalk between autophagy, inflammasomes and apoptosis could be an important underlying mechanism involved in the beneficial effects of exercise in the elderly, and reinforce the idea that regular physical exercise is one of the most potent anti-aging treatments.

## METHODS

### Participants

Twenty-six healthy participants (7 males, 19 females; age range: 65-78) volunteered to participate in the study. The inclusion criteria specified not to take any medication known to affect the inflammatory status in the 6 months prior to or during the study. Subjects which had received previous medication for dyslipidemia, hypertension and diabetes mellitus were also excluded for the study. Moreover, none of the female participants were taking any hormonal treatment, either before or at the time of the study. Participants did not have any experience in resistance exercise training, and they were asked to maintain their physical activity routines during the study period. For the main experimental part, before any other activity, a medical screening including anthropometric analysis, the physical activity readiness questionnaire (PAR-Q), a risk factor quiz, blood pressure measurements, and a basal electrocardiogram test were performed in all participants. Subjects were randomly assigned to a training group (n=16) or to a control group (n=10). Age, height, weight and body mass index were: 69.2 ± 1.0 yr, 158.3 ± 1.9 cm, 68.4 ± 2.4 kg and 26.8 ± 0.5 kg/m^2^ respectively for TG, and 70.2 ± 0.8 yr, 159.2 ± 2.0 cm, 68.1 ± 2.8 kg and 27.3 ± 0.6 kg/m^2^ respectively for CG. Participants from TG followed an 8-week resistance exercise training program, whereas the control group kept their normal daily routines. All volunteers were informed of the objectives and possible risks of the intervention before individual written consent for participation was obtained. The study followed the principles of the Declaration of Helsinki, and the local ethics committee approved all procedures.

### Experimental design and maximal strength assessment

This study was completed in 10 weeks. Subjects performed a resistance exercise training program during 8 weeks and baseline data were collected one week prior (Pre) and after (Post) the exercise protocol. In the weeks 1 and 10, and after a standardized 10-min warm up on a cycle ergometer (Tunturi F35, Tunturi^®^, Turku, Finland), maximal voluntary isometric contraction test was carried out in a 45°-inclined leg press device (Gervasport, Madrid, Spain) at 110° knee flexion, and in a biceps curl bench device (Gervasport) at 90 elbow flexion. Maximal strength was registered by a strain gauge (Globus Ergometer, Globus, Codogne, Italy). After ∼30 min of rest, one repetition maximum test was performed in the same leg-press and biceps curl bench previously described, and in a seated pec deck machine (BH Fitness Nevada Pro, BH, Vitoria, Spain).

### Resistance exercise training

Subjects from the TG completed 16 resistance exercise training sessions over 8 weeks (2 sessions per week), with at least 48 h between sessions. After a 10-min warm-up on a cycle ergometer, participants performed 3 different exercises, i.e. leg press, biceps curl and pec deck, in the same exercise devices described above. The number of repetitions per set, as well as the load for the three exercises were progressively increased as follow: 3×8, 3×10 and 3×12 at 60% of 1RM during weeks 1, 2 and 3 respectively; 3×8, 3×10 and 3×12 at 70% of 1RM during weeks 4, 5 and 6, respectively; and 3×8 and 3×10 at 80% of 1RM during weeks 7 and 8, respectively.

### Venus blood sampling

Using Vacutainer™ system (BD, Franklin Lakes, NJ) with EDTA as anticoagulant, blood samples (30 ml) were obtained in the early morning in the fasted state from the brachiocephalic vein, 5-6 days before and after the training period. PBMCs were isolated from the whole blood by density gradient centrifugation on Ficoll separating solution (Biochrom AG, Berlin, Germany) [47]. PBMCs subpopulation count was carried out using a particle counter (Beckman-Coulter, Miami, FL, USA). PBMCs subpopulations were measured before and after training with no difference between time points (data not shown).

### Western blot analysis

PBMCs were homogenized in 150 μl buffer containing 0.25 mM sucrose, 1 mM EDTA, 10 mM Tris and a protease inhibitor cocktail (Sigma-Aldrich, St. Louis, MO, USA) with an ultrasonic processor (UP100H, Hielscher, Teltow, Germany). Protein content of each sample was measured by the technique described by Bradford [48]. Samples containing 50 μg of protein were separated by SDS-PAGE on 8% (beclin-1, Atg12, Atg16, LAMP-2, phospho-ULK-1and β-actin), 9% NLRP3 and p62/SQSTM1) or 12% (Bad, Bcl-2, Bcl-xL, LC3II/I, cleaved caspase-3, procaspase-1 and caspase-1) SDS-polyacrylamide gels. Separated proteins were transferred to PVDF membranes and then, non-specific binding was blocked by pre-incubation of the membranes in 5% non-fat milk-PBS for 30 min at 37°C. After that, incubation with specific primary antibodies was performed overnight at 4°C. Antibodies against Atg12 (21 kDa, Ref. sc-68884), Atg16 (63/71 kDa, Ref. sc-70133), beclin-1 (60 kDa, Ref. sc-11427), LAMP-2 (120 kDa, Ref. sc-5571), Bad (23 kDa, Ref. sc-8044) and Bcl-xL (30 kDa, Ref. sc-1041) were purchased from Santa Cruz Biotechnology, CA, USA. LC3I/II (14-16 kDa, Ref. 12741S), p62/SQSTM1 (60 kDa, Ref. 5114), phospho-ULK-1 (Ser^757^) (140-150 kDa, Ref. 6888), NLRP3 (110 kDa, Ref. 15101), cleaved caspase-3 (17-19 kDa, Ref. 9662) and procaspase-1 and caspase-1 (20 and 50 kDa, respectively, Ref. 2225) were purchased from Cell Signaling Technology®, Beverly, MA, USA. Bcl-2 (25-26 kDa, Ref. ab692) was purchased from Abcam^®^, Cambridge, UK, USA, and β-actin (42 kDa, Ref. A5060), which served as control protein, was purchased from Sigma-Aldrich, St Louis, MO, USA. Bound primary antibody was detected using a peroxidase-conjugated secondary antibody (Dako, Glostrup, Denmark) and an enhanced chemiluminescence-HRP kit (Luminol Reagent Santa Cruz Biotechnology). The density of the specific bands was quantified with an imaging densitometer (Image J, Bethesda, MD, USA).

### Statistical analysis

Values are presented as mean ± standard error of means (SEM). Post-training values were normalized to pre-training values. Saphiro-Wilk test was used to verify normal data distribution. All data were analyzed using a two-way analysis of variance (ANOVA) with repeated measures for group (GG and TG) and time (pre and post). Bonferroni analysis was used to compensate for multiple post hoc comparisons. Differences were considered significant when p<0.05. All statistical analyses were performed using SPSS version 18 (SPSS Inc., Chicago, IL, USA).
